# Obstacle Avoidance of Multi-Sensor Intelligent Robot Based on Road Sign Detection

**DOI:** 10.3390/s21206777

**Published:** 2021-10-12

**Authors:** Jianwei Zhao, Jianhua Fang, Shouzhong Wang, Kun Wang, Chengxiang Liu, Tao Han

**Affiliations:** 1 School of Mechatronic Engineering, China University of Mining and Technology, Beijing 100089, China; zhaojianwei@cumtb.edu.cn (J.Z.); ZQT1900401034G@student.cumtb.edu.cn (K.W.); ZQT1900401022G@student.cumtb.edu.cn (C.L.); ZQT1900401010G@student.cumtb.edu.cn (T.H.); 2 Beijing Special Engineering and Design Institute, Beijing 100028, China; 18901181265@189.cn

**Keywords:** intelligent robot, multi sensor, Adaboost, obstacle avoidance, object detection

## Abstract

The existing ultrasonic obstacle avoidance robot only uses an ultrasonic sensor in the process of obstacle avoidance, which can only be avoided according to the fixed obstacle avoidance route. Obstacle avoidance cannot follow additional information. At the same time, existing robots rarely involve the obstacle avoidance strategy of avoiding pits. In this study, on the basis of ultrasonic sensor obstacle avoidance, visual information is added so the robot in the process of obstacle avoidance can refer to the direction indicated by road signs to avoid obstacles, at the same time, the study added an infrared ranging sensor, so the robot can avoid potholes. Aiming at this situation, this paper proposes an intelligent obstacle avoidance design of an autonomous mobile robot based on a multi-sensor in a multi-obstruction environment. A CascadeClassifier is used to train positive and negative samples for road signs with similar color and shape. A multi-sensor information fusion is used for path planning and the obstacle avoidance logic of the intelligent robot is designed to realize autonomous obstacle avoidance. The infrared sensor is used to obtain the environmental information of the ground depression on the wheel path, the ultrasonic sensor is used to obtain the distance information of the surrounding obstacles and road signs, and the information of the road signs obtained by the camera is processed by the computer and transmitted to the main controller. The environment information obtained is processed by the microprocessor and the control command is output to the execution unit. The feasibility of the design is verified by analyzing the distance acquired by the ultrasonic sensor, infrared distance measuring sensors, and the model obtained by training the sample of the road sign, as well as by experiments in the complex environment constructed manually.

## 1. Introduction

With the development of artificial intelligence technology, mobile robots are widely used in intelligent factories, modern logistics, security, precision agriculture and other aspects [1,2,3,4]. Wheeled mobile robots have been widely used in storage and transportation fields. The focus of this research is to avoid obstacles in complex environments while the path is optimal. The most important thing to realize the autonomous motion control of a mobile robot is to obtain the information of the surrounding environment and transfer it to the main controller to convert it into control command, so as to ensure that the robot can safely and stably avoid all obstacles while moving to the destination, which can be achieved when the mobile robot has a strong perception system. Different types of sensors are required for different information, including proprioceptive sensors that measure information such as joint angle and wheel speed; and exteroceptive sensors that sense external data such as sound, light and distance [5,6,7,8,9,10]. The sensing technologies of mobile robots include passive sensing based on multiple cameras, stereo vision and infrared cameras and active sensing using lidar and sonar sensors to detect dynamic or stationary obstacles in real time [11]. Laser ranging is used to analyze the wheel skid of the four-wheel sliding steering mobile robot. Some other studies have proposed target tracking of wheeled mobile robots based on visual methods [12,13].

For an unknown environment, sensors are usually used for intelligent obstacle avoidance and path planning. The early method of obstacle avoidance and path planning is to detect the stickers on the ground by infrared ray for navigation. This method can only be used in a known environment. In Ref. [14] Jiang et al. [15] utilized six ultrasonic sensors to capture relative information about of ambient wheeled robots and to identify a parking space for automatic parking. In 1995, Yuta and Ando [16] installed ultrasonic sensors on the front of a robot and in various locations on the left and right sides. In Refs. [17,18] multiple ultrasonic data were used to create the map of the surrounding environment or establish the surface shape of obstacles.

At present, the research on an obstacle avoidance robot is mostly about the motor driving principle, motor speed regulation scheme and ranging principle, and the research on obstacle avoidance is also about obstacle avoidance. Few people study mobile robots when they encounter pits during automatic travel. In this paper, ultrasonic sensor information, infrared distance measuring sensors information and camera information are fused. After solving the above problems, the function of road sign recognition is also introduced, which allows the mobile robot to make accurate movement based on the traffic sign information.

## 2. Establishing Kinematic Model

The object of this paper is a wheeled sliding steering mobile robot, which is driven independently by four symmetrical wheels and has both rolling and sliding in the movement process, and its mechanical structure is simple and has high flexibility. The car body has no steering gear and relies on changing the left and right wheel speeds to make the wheels skid, achieving a different radius steering or even zero radius steering. However, due to the nonlinear, time-varying, multi-variable and strong coupling characteristics of the system, its motion is more uncertain than that of the car with a steering device. So, it is necessary to build a kinematic model for the system instead of using a simple kinematic model to represent its motion characteristics.

**Figure 1:**
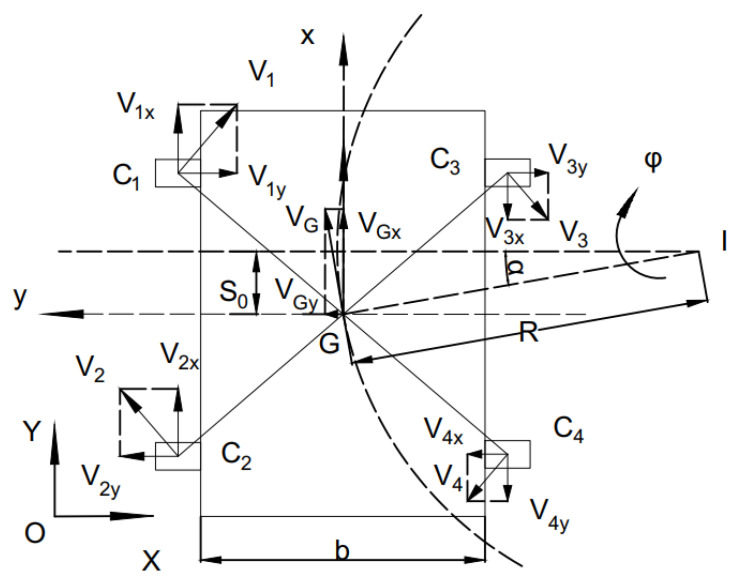
Kinematic model.

Figure 1 shows the operational model of the robot studied in this study. Without considering the mass transfer, the following assumptions are made for the model:

1. The vehicle body is completely symmetrical and the center of mass coincides with the center of assembly;
2. The car moves in plane motion.

We established the fuselage coordinate system Ob and the ground coordinate system XOY, where the fuselage coordinate system moves with the vehicle.

In fuselage coordinates system:

(1) $$\{\begin{array}{c} v_{Gx}=v_{G}cos\alpha \\ v_{Gy}=v_{G}sin\alpha \end{array}$$

In the process of moving, the relative sliding between the mobile robot and the ground is inevitable. We used the slip rate to describe the wheel slip and its calculation formula is:

(2) $$s_{i}=\frac{w_{i}r-v_{ix}}{w_{i}r}\times 100\%$$

$w_{i}$ is the rotational speed of wheel *i*;

$v_{ix}$ is the x-direction partial velocity of the center of wheel *i*.

When $s_{i}>0$, that is, the wheel linear velocity is greater than the wheel center velocity, the frictional force between the wheels and the ground is the driving force, and then the slip occurs.

When $s_{i}<0$, that is, the wheel linear velocity is less than the wheel center velocity, the frictional force between the wheels and the ground is the braking force and slippage occurs.

When $s_{i}=0$, that is, the linear speed of the wheel is equal to the speed of the wheel center, the robot is in a complete rolling state.

We defined the side near the center of rotation as the inside side and the other side as the outside side. When the robot turns, the inner wheel slips and the outer wheel slips. The instantaneous center of the contact point between the wheels on both sides and the ground is equal to the y coordinate of the rotating center.

When the robot rotates, the longitudinal velocity at the center of the same-side wheel is equal:

(3) $$w_{1}r\left(1-s_{1}\right)=w_{2}r\left(1-s_{2}\right)$$

(4) $$w_{3}r\left(1-s_{3}\right)=w_{4}r\left(1-s_{4}\right)$$

Because the wheels on the same side rotate at the same speed, so:

(5) $$\;s_{1}=s_{2}=s_{l},\;s_{3}=s_{4}=s_{r}$$

Formula (4) shows the relationship between linear velocity, attitude angular velocity and left and right wheel rotation speed:

(6) $$\{\begin{array}{c} v=\frac{w_{l}r+w_{r}r}{2}\\ \dot{\varphi }=\frac{w_{r}r-w_{l}r}{b} \end{array}$$

Since there is sliding, the linear velocity is represented by the longitudinal velocity of the wheel center, while the lateral velocity can be represented by the sideslip angle, so formula (5) can be obtained.

(7) $$\left[\begin{array}{c} v_{Gx}\\ v_{Gy}\\ \dot{\varphi } \end{array}\right]=\frac{r}{2}\left[\begin{array}{cc} 1-s_{l} & 1-s_{r}\\ tan\alpha \left(1-s_{l}\right) & tan\alpha \left(1-s_{r}\right)\\ -\frac{2\left(1-s_{l}\right)}{b} & \frac{2\left(1-s_{r}\right)}{b} \end{array}\right]\left[\begin{array}{c} w_{l}\\ w_{r} \end{array}\right]$$

Through coordinate system transformation, the kinematics equation of the mobile robot in the ground coordinate system XOY can be expressed by formula (6).

(8) $$\left[\begin{array}{c} \dot{X}\\ \dot{Y}\\ \dot{\theta } \end{array}\right]=\left[\begin{array}{ccc} cos\theta & sin\theta & 0\\ -sin\theta & cos\theta & 0\\ 0 & 0 & 1 \end{array}\right]\left[\begin{array}{c} v_{Gx}\\ v_{Gy}\\ \dot{\varphi } \end{array}\right]$$

The meanings of parameters in the formula are shown in Table 1.

## 3. System Structure of the Mobile Robot

**Figure 2:**
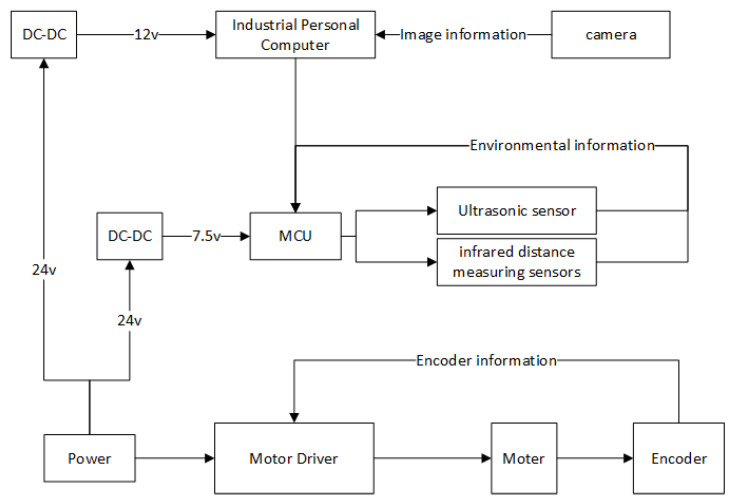
Composition of mobile robot system.

The control system of the mobile robot is composed of the power module, main controller, industrial personal computer (IPC), detection module and drive module. The power supply module is responsible for the energy supply of the whole system. Since the voltage of each module is different, the voltage and regulation are performed by the up/down module. The voltage of the ultrasonic sensor and the infrared distance measuring sensors need only be provided by the controller. The detection module comprises an ultrasonic sensor, an infrared distance measuring sensor and a camera. The detection module is mainly used for detecting the surrounding environment, and feeds the detected environment information back to the main controller. The camera data shall be first handed over to the IPC, and then fed back to the main controller after processing by the IPC. The main controller processes the environment information. The drive module consists of a motor driver as well as an encoder motor. The motor driver controls the speed of the encoder motor. The motor encoder detects the speed of the motor and feeds it back to the driver for closed-loop control. The main controller is responsible for the information processing of the system using the Arduino Mega 2560. The detection system detects that the environment information is fed back to the main controller, and the main controller processes the information. The driving system is driven according to the environment information to control the movement speed and attitude of the robot, so as to avoid obstacles in the range of activity. Figure 2 shows the system composition of the mobile robot.

## 4. Detection System

In the research of obstacle avoidance of a mobile robot, the processing of the surrounding environment information is especially important. The environment is dynamic and unknown in real life. At the same time, in some environments, there are signs that require the robot to move in the specified direction. It is important that the robot moves safely to its destination in a complex location. By selecting appropriate sensors to collect and analyze environmental information, the robot can realize the above functions. In this design, the HC-SR04 ultrasonic sensor, GP2YA02 and USB driver-free camera are selected for the components of the detection system.

### 4.1. Sensor Layout

**Figure 3:**
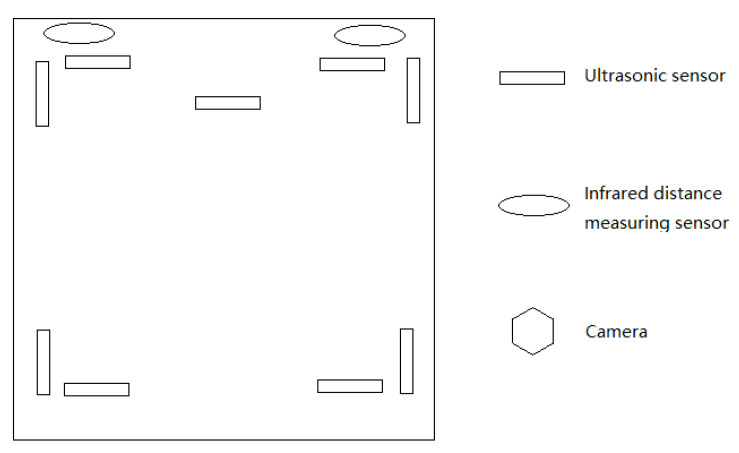
Sensor Layout.

In order to make the robot work normally in both static and dynamic environments, nine ultrasonic sensors, two infrared distance measuring sensors, and a USB driverless camera were installed on the robot body. An ultrasonic sensor was used to detect obstacle information of the surrounding bulge; an infrared distance measuring sensor was positioned between two wheels in front of the bottom wheel for detecting the ground pit; a camera was used to detect the road sign information. The information detected by the sensor is transmitted to the main controller for processing, and a command is sent to the motor driver to control the robot for corresponding movement. The sensor layout of the mobile robot is shown in Figure 3. 

### 4.2. Target Detection Based on Adaboost Algorithm

#### 4.2.1. Sample Pretreatment

**Figure 4:**
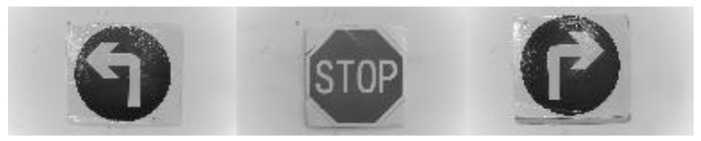
Positive sample picture.

**Figure 5:**
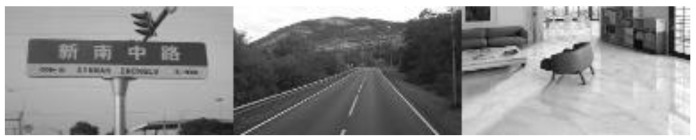
Negative Sample Picture.

The training sample is divided into a positive sample and negative sample, the positive sample is a road sign sample picture and the negative sample is any other picture. In this paper, 1000 positive samples and 2000 negative samples were selected, and samples were grayed and normalized to 128 × 72 gray scale as to form a training sample set, so as to avoid different pictures to calculate a different number of features. The picture shown in Figure 4 is the sample of the three road signs to be trained on separately. Figure 5 is the picture of the negative sample.

#### 4.2.2. CascadeClassifier Training Based on Adaboost

The Adaboost algorithm is an adaptive boosting algorithm. The basic idea of Adaboost is to use weak classifier and sample space of different weight distribution to build a strong classifier [19,20,21,22]. The Adaboost algorithm synthesizes a strong CascadeClassifier with a strong classification ability by superposing a large number of simple CascadeClassifiers with general classification ability. A strong CascadeClassifier is formed by selecting weak CascadeClassifiers with the best resolution performance and the least error. The principle is to carry out T cycle iteration, select an optimal and weak CascadeClassifier each time, then update the sample weight, reduce the weight of correctly resolved samples, and increase the weight of incorrectly resolved samples. The specific algorithm is as follows [23,24,25]:

Step 1: given a set of data sets for training:

(9) $$\{\{\;x_{1},y_{1}\},\left\{\;x_{2},y_{2}\right\},\ldots ,\left\{\;x_{n},y_{n}\right\}\},$$

where $x_{i}\;$ is the input training sample images, $y_{i}$ is the result of classification and $\;y_{i}\in \left[0,\;1\right]$ is 1 sample, 0 means a negative sample;

Step 2: specifies the number of loop iterations;

Step 3: initializes the weight of the sample:

(10) $$w^{1}=\left\{w_{1,1}\ldots ,w_{1,N}\right\},w_{1,j}=d\left(i\right),$$

where d(i) is the distribution probability used to initialize the strictly impossible;

Step 4: t=1,2,…,T (T is the number of training times, which determines the number of final weak CascadeClassifiers):

(1): Initialization weight:

(11) $$p^{t}=p_{t1},\ldots ,p_{tN},$$

(2): The initial sample is trained by a learning algorithm to obtain a weak CascadeClassifier.

(12) $$h_{t}:X\to \left[0,\;1\right],$$

(3): The error rate of each weight under the current weight is found:

(13) $$\varepsilon_{t}=\sum_{i=1}^{N}p_{t,i}\left|h_{t}\left(X_{i}\right)-y_{i}\right|,$$

the CascadeClassifier with the smallest error rate from the obtained weak CascadeClassifier is selected and added to the strong CascadeClassifier

(4): Weight:

(14) $$\omega_{t+1,i}=\omega_{t,i}\beta^{1-\left|h_{t}\left(x_{i}\right)-y_{i}\right|},$$

If the sample of i is classified correctly:

(15) $$\left|h_{t}\left(x_{i}\right)-y_{i}\right|=0,$$

Otherwise:

(16) $$\left|h_{t}\left(x_{i}\right)-y_{i}\right|=1,$$

where:

(17) $$\alpha_{t}=\frac{\varepsilon_{t}}{1-\varepsilon_{t}},$$

(5): After passing the T wheel, the strong CascadeClassifier obtained is:

(18) $$H\left(x\right)=\{\begin{array}{c} 1\;\;\;\;\;\;\overset{T}{\underset{t=1}{\sum }}\alpha_{t}h_{t}\left(x\right)\geq \frac{1}{2}\overset{T}{\underset{t=1}{\sum }}\alpha_{t}\\ 0\;\;\;\;\;\;\;\;\;\;\;\;\;\;\;\;\;\;\;\;\;\;\;\;\;\;\;\;\;\;\;\;\;\;\;\;\;\;\;\;\;other \end{array},$$

where

(19) $$\alpha_{t}=\log\frac{1}{\beta_{t}},$$

#### 4.2.3. Road Sign Identification Process

**Figure 6:**
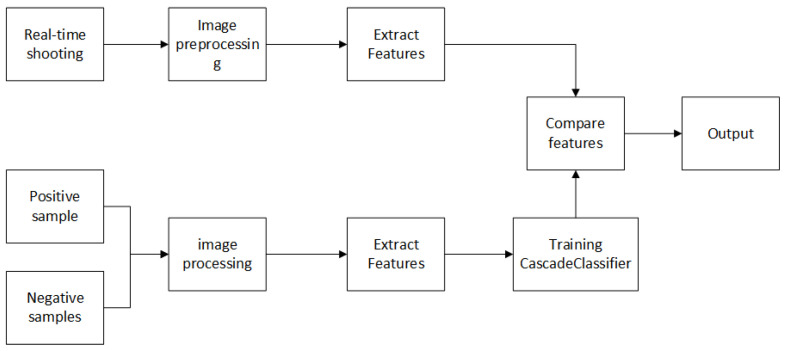
Identification flow chart.

Figure 6 shows the identification flow chart. The whole process can be divided into two steps: training and identification. In the training process, the Haar features are used to extract the features of a large number of road sign samples, and then the Adaboost algorithm is used to select the effective features to form the CascadeClassifier. In the recognition process, the key features of the samples to be identified are extracted first, and then the features and the trained CascadeClassifier are used for road sign recognition.

## 5. Obstacle Avoidance Strategy for Mobile Robots

**Figure 7:**
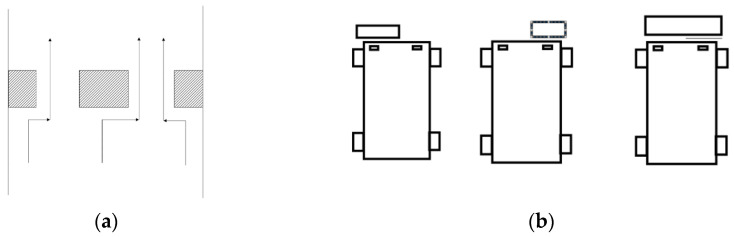
Schematic diagram of obstacle, (**a**) is the raised obstacle on the ground; (**b**) is a sunken obstacle on the ground.

**Figure 8:**
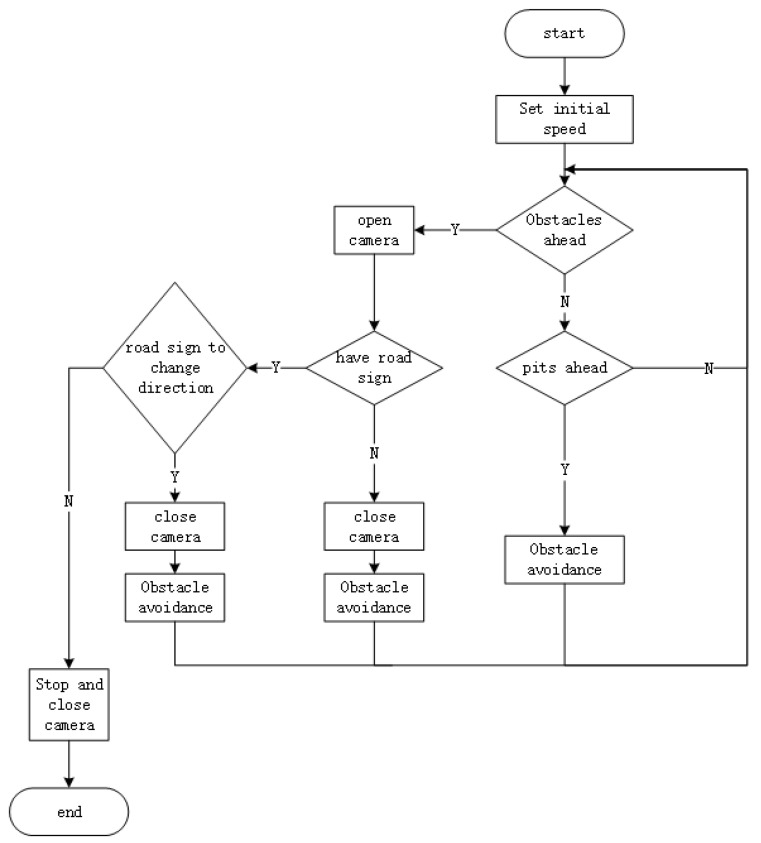
Block diagram of obstacle avoidance program.

In this study, the obstacle avoidance is mainly realized in the following two situations, namely, obstacle avoidance for a ground bulging obstacle and obstacle avoidance for a ground sag obstacle. Figure 7 shows the schematic diagram of obstacles and obstacle avoidance routes in this study. We made the following assumptions about obstacles:

1. Ground raised obstacles and a ground pit will only meet the conditions shown in Figure 8;
2. Ground raised obstacles and a ground pit do not appear simultaneously;
3. The road sign is present on a raised obstacle;
4. The ground pit width is less than the wheel spacing of the robot;
5. There is a stop road sign at the destination that prompts stop.

Figure 8 shows a block diagram of the motion of a mobile robot. During the operation of the intelligent vehicle, first initialize the data and give the vehicle an initial forward speed; then start the ultrasonic sensor in front and the infrared distance measuring sensors; if the ultrasonic sensor detects an obstacle, stop and start the camera to judge whether there is a road sign indication; if there is a road sign indication, avoid the obstacle according to the road sign indication; if there is no road sign, avoid the obstacle autonomously according to the built-in program; if the infrared distance measuring sensors detect a ground pit, use the built-in program to perform the corresponding movement of avoiding the ground pit. 

When the robot avoids obstacles, it needs to leave a certain space so that the robot can turn safely. Since the length and width of the robot are both 70 cm, the distance between its rotating center and the furthest point is about 50 cm. The safe distance is set as 60 cm because the robot has deviation when rotating. When the distance measured by the sensor in the front is equal to 60 cm, it indicates that there is an obstacle in front. At this time, open the camera to detect whether there is a road sign. If the road sign is detected, the obstacle should be avoided in the direction indicated by the type of road sign. If no road sign is detected, turn off the camera and perform obstacle avoidance. The reason cameras are used only when obstacles are detected is that road signs are fixed to the surface of obstacles on the ground, and because there is no other way of ranging other than by ultrasonic sensors, the cameras cannot determine the distance of road signs once they are detected. Therefore, the ultrasonic sensor detects the obstacle and determines the distance of the obstacle, and then turns on the camera to determine whether there is a road sign on the obstacle and the distance of the road sign.

The obstacle avoidance movement of raised obstacles on the ground is as follows: when the distance of obstacles detected by the ultrasonic sensor on the front side is 60 cm, the left and right ultrasonic waves start to detect for obstacles. If the distance measured by the ultrasonic on the left is greater than that measured by the ultrasonic sensor on the right, it turns to the left. At this time, the speed of the left wheel and the speed of the right wheel are reversed and the left wheel reverses. If the distance measured by the ultrasonic on the right is greater than that measured by the ultrasonic sensor on the left, it turns to the right. At this time, the speed of the left wheel is in reverse with that of the right wheel, and the left wheel is turning positively. After successful turning, the robot drives forward until it is out of the range of the obstacle. At this point, the robot turns in the opposite direction of the previous turning direction, and then continues driving and finally leaves the obstacle. When the robot leaves the obstacle range, the detection value of the ultrasonic sensor on the side of the robot is greater than 60 cm.

The obstacle avoidance movement of the ground pits is as follows: the distance between the infrared sensor and the ground is 6 cm, and the distance between the chassis and the ground is 3 cm, so the safe distance is less than 9 cm, which is set to 8 cm in this experiment. So, the distance detected by the infrared ranging sensor is greater than 8 cm to avoid the past. When the detection distance of the left infrared sensor is greater than or equal to 8 cm, the left wheel slows down, and the right wheel accelerates to the left to avoid the pit. When the detection distance of the right infrared sensor is greater than or equal to 8 cm, the right wheel slows down, and the right wheel accelerates to the right to avoid the pit. When pits are detected on both sides, the vehicle stops and waits for manual movement.

## 6. Experiment and Analysis

**Figure 9:**
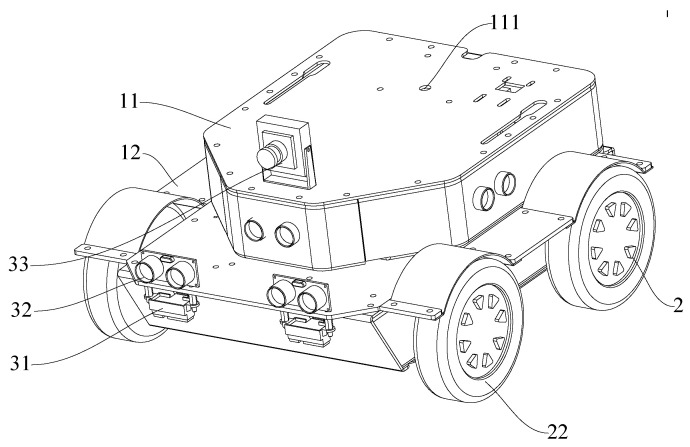
Physical prototype.

Figure 9 is the physical prototype used in the experiment, and Table 2 is the mark in the physical prototype diagram. The prototype includes a vehicle body, drive assembly, detection assembly and control assembly. The drive assembly includes a drive member for driving a wheel to rotate relative to a vehicle body and a wheel. A plurality of detection assemblies is connected to the vehicle body, a portion of the detection assemblies (ultrasonic sensors) are used to detect obstacles around the vehicle body, a portion of the detection assemblies (infrared sensors) are used to detect obstacles at the lower end of the vehicle body, and a portion of the detection assemblies (cameras) are used to detect road sign information. The control assembly is connected to a drive member and a detection assembly to receive a signal from the detection assembly and control the drive member to drive a wheel to turn when the detection assembly detects an obstacle to avoid the obstacle.

### 6.1. Sensor Ranging Experiment

**Figure 10:**
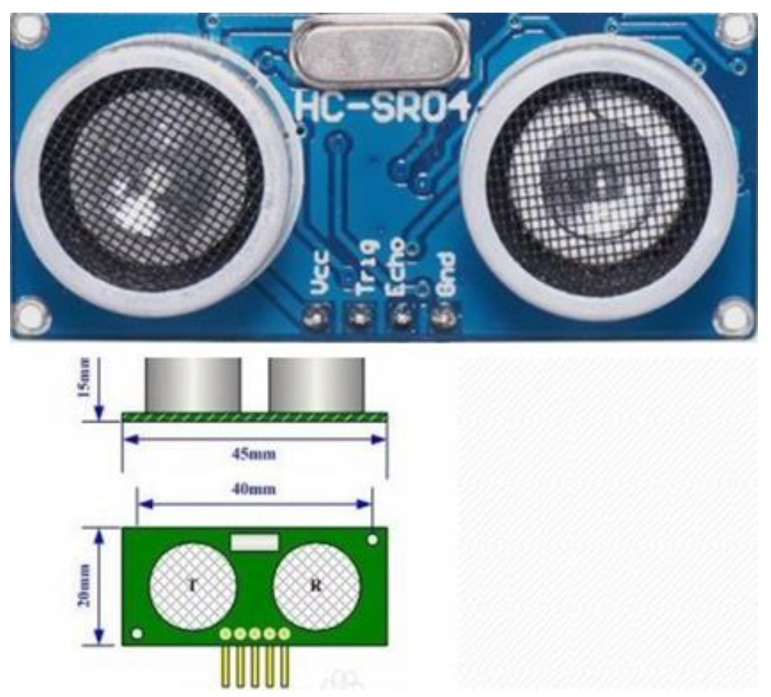
Ultrasonic transducer.

Figure 10 is an ultrasonic sensor. The HC-SR04 ultrasonic ranging module can provide 2 cm–450 cm non-contact ranging function, ranging accuracy up to 3 mm; the module includes an ultrasonic transmitter, receiver and control circuit.

The ultrasonic module has four pins:, Trig (control end), Echo (receiving end), GND; VCC and GND are connected to 5 V power supply, Trig (control end) controls the ultrasonic signal sent, and Echo (receiving end) receives the reflected ultrasonic signal.

**Figure 11:**
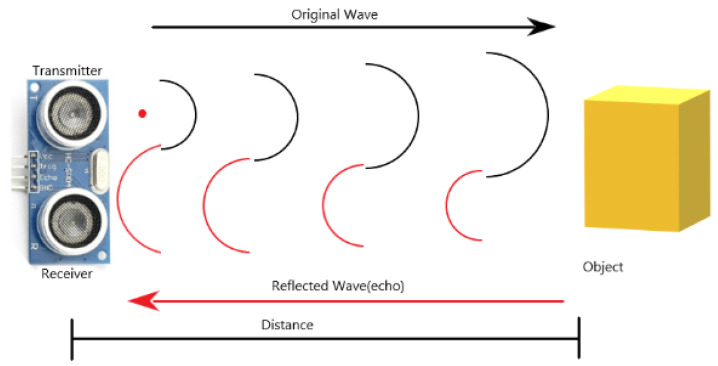
Ranging principle.

Figure 11 is the principle of ultrasonic sensor ranging. The ultrasonic sensor ranging is based on the reflection characteristics of the ultrasonic sensor. The transmitter end of the ultrasonic sensor emits a beam of ultrasonic wave, and at the same time it starts timing, and the ultrasonic wave is transmitted in the medium at the same time. Because sound waves have reflective properties, they bounce back when they encounter obstacles. When the receiving end of the ultrasonic sensor receives the reflected ultrasonic wave back, it stops the timing. The propagation medium in this study is air, and the propagation speed of sound in air is 340 m/s. According to the recorded time t, the distance S between the launching position and the obstacle can be calculated according to the formula S = 340 t/2.

**Figure 12:**
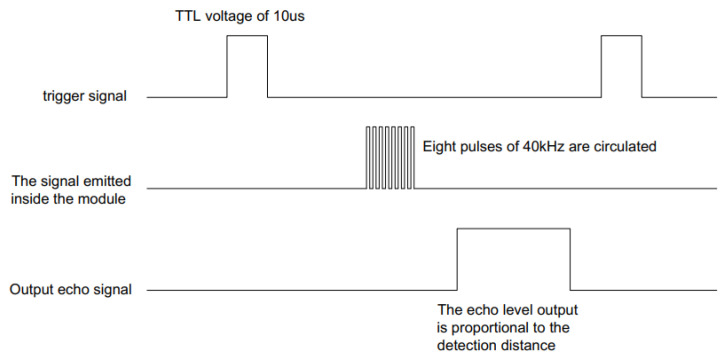
Sequence diagram of ultrasonic sensor.

**Figure 13:**
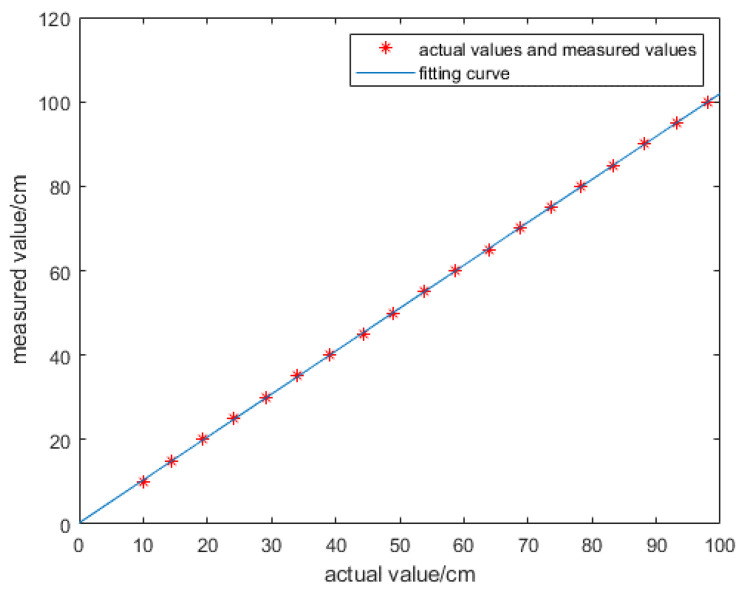
Curve after fitting.

Figure 12 is a sequence diagram of an ultrasonic sensor. As shown in Figure 13, a pulse trigger signal of more than 10 needs to be provided, for 8 cycle levels of 40 kHz to be emitted inside the module and the echo to be detected. Once the echo signal is detected, the echo signal is output. The pulse width of the echo signal is proportional to the measured distance. The formula for calculating distance can be obtained from the time interval between the transmitting signal and receiving echo signal: distance = high level time * sound speed/2. In order to avoid the influence of the transmitting signal on a recall signal, the measurement period is above 60 ms.

An ultrasonic sensor was used to measure the value of distance from the object. The test distance and actual distance of ultrasonic sensor obtained through multiple experiments are shown in Table 3.

In order to improve the accuracy of the distance value measured by the sensor, use MATLAB to fit the curve of each distance value in the table with the least square method, and the fitting curve is y=ax+b, where a=1.0171,b=0.262, as shown in Figure 13. In Figure 13 the longitudinal axis shows the measured value, and the horizontal axis shows the actual values, the unit is cm. Table 4 shows the actual value and the value after fitting. It can be seen from the table that the error is very small

### 6.2. Road Sign Detection Experiment

**Figure 14:**
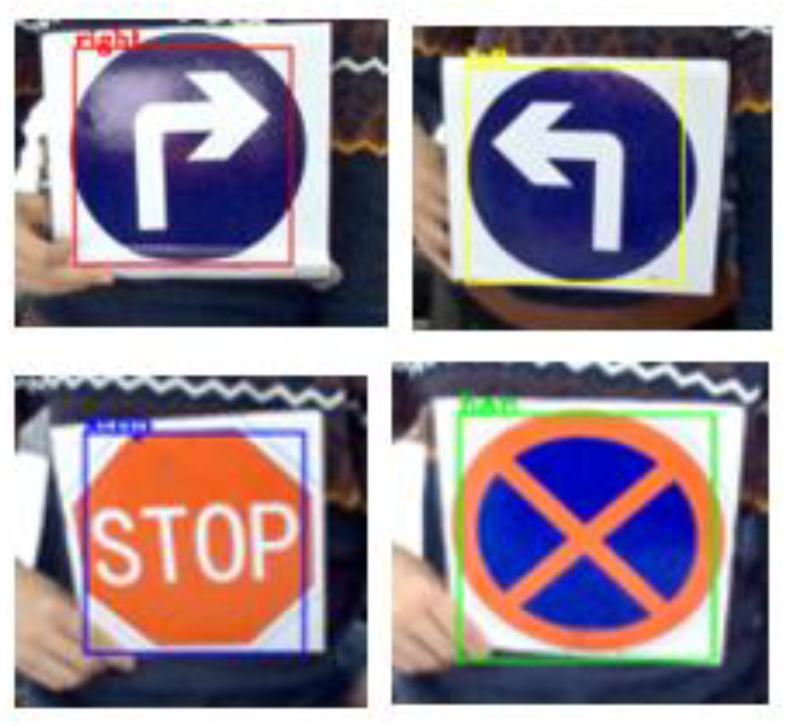
Identification effect of road signs.

**Figure 15:**
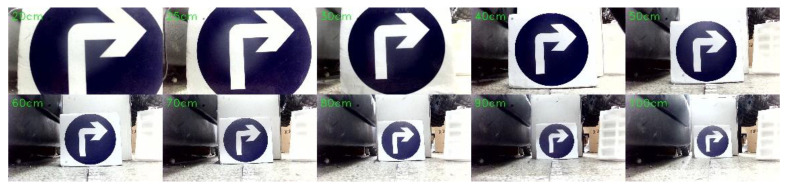
Road sign detection environment.

**Figure 16:**
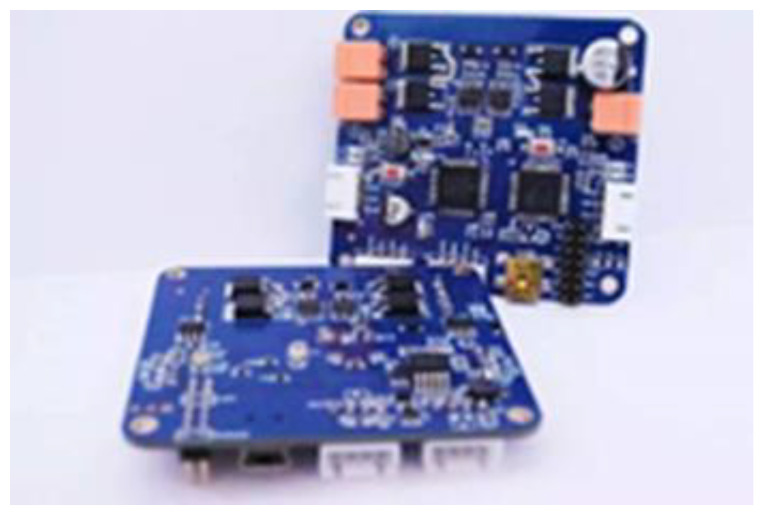
Motor driver.

Figure 14 shows the experimental effect diagram of detecting the actual road sign using the camera and the model after training. Figure 15 shows the actual environment of the detection experiment, and the background of the actual detection environment is not pure color, which can meet the requirements of the robot. In Figure 16, from left to right, and from top to bottom, the test distances are 10 cm, 20 cm, 30 cm, 40 cm, 50 cm, 60 cm, 70 cm, 80 cm, 90 cm, and 100 cm. In this study, experiments were conducted on road signs at different distances. The test results show that in the detection environment where the camera is 20 cm away from the road sign and 25 cm away from the road sign, the road sign cannot all be in the field of view due to the influence of the camera parameters, it is only partially in the field of view. Table 5 and Table 6 are the experimental data of road sign recognition. From these two tables, it can be concluded that the success rate of recognition is lower when the distance between the camera and the road sign is less than 25 cm. The success rate of recognition is higher when the distance between the camera and the road sign is greater than or equal to 30 cm, reaching an average of 99.625%, which can meet the requirements of accurate obstacle avoidance.

### 6.3. Physical Test

**Figure 17:**
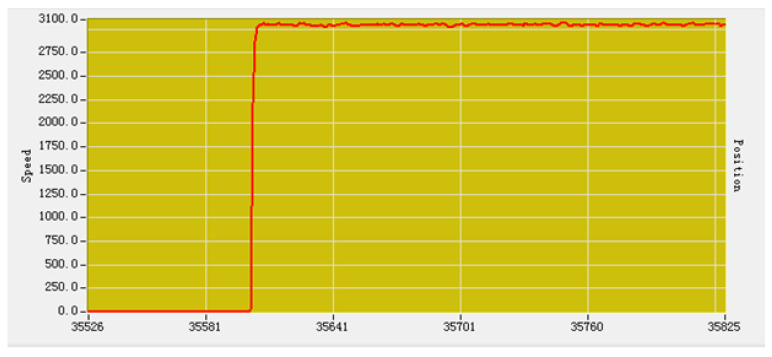
Motor speed curve.

Figure 16 represents a neurons intelligent PID motor drive module with a built-in controller capable of PID computation, trapezoidal control, and dc motor movement driven by a drive circuit on the circuit board. Through the serial port it can send 8 B of command to control the positive and negative movement of the dual motor. Figure 17 is the motor speed curve after PID adjustment. It can be observed from the figure that when the speed of the motor increases from 0 to the maximum speed suddenly, the overshoot is very small and the curve reaches dynamic equilibrium in a very short time.

**Figure 18:**
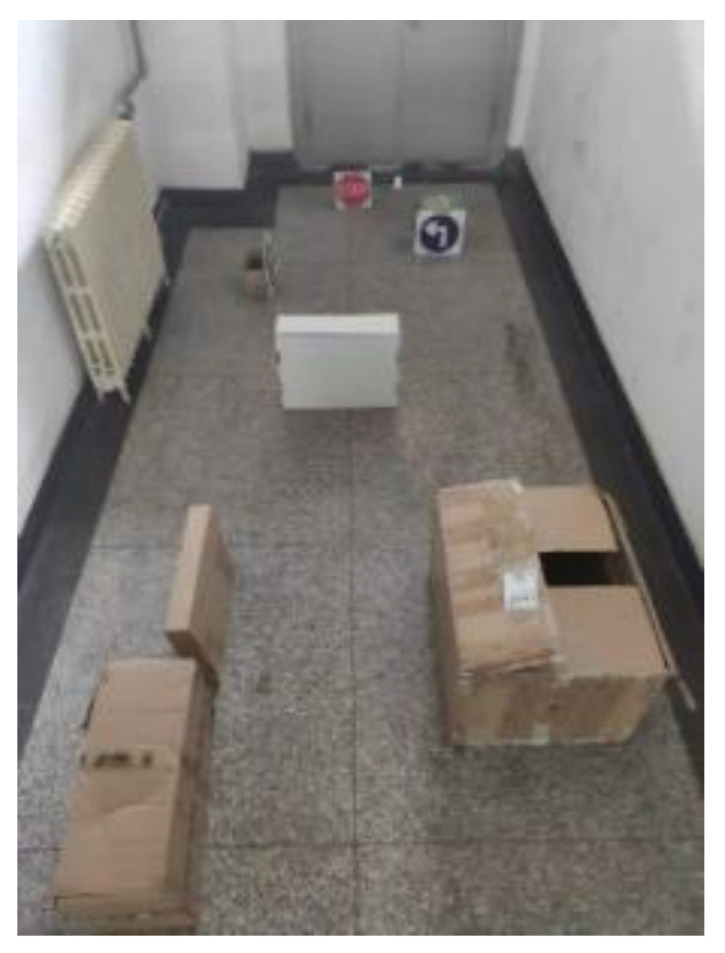
Experimental Environment.

**Figure 19:**
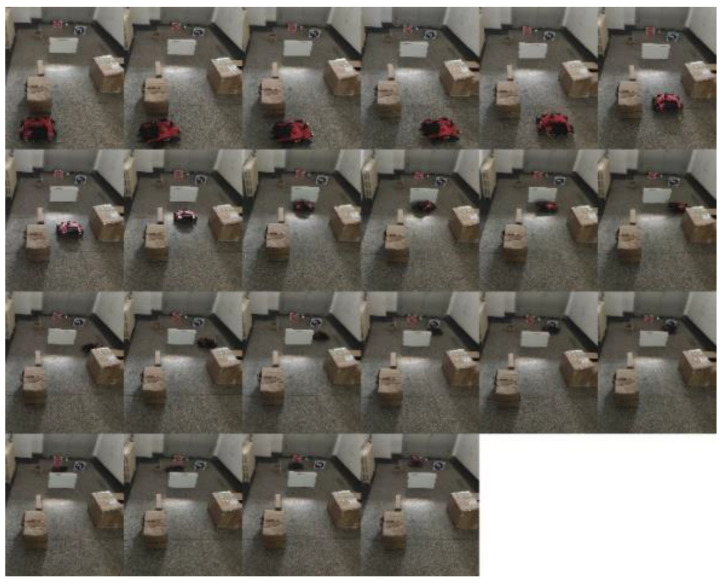
Physical prototype experiment.

**Figure 20:**
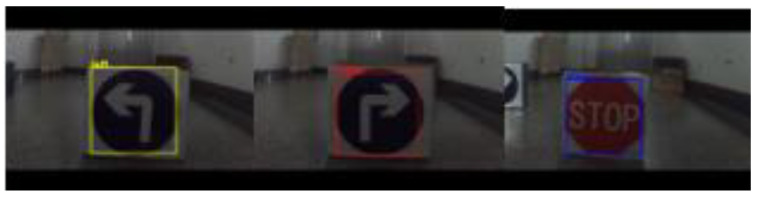
Real-time picture of road sign detection.

Figure 18 shows the artificially constructed experimental environment, including the environment of obstacles and road signs. The obstacle avoidance experiment of the physical prototype was conducted in the constructed experimental environment, and the experimental results are shown in Figure 19 The experimental results show that this method can successfully identify road signs and realize autonomous obstacle avoidance in complex environments. Figure 20 shows the real-time picture of road sign detection (not the experimental environment as shown in Figure 19).

## 7. Conclusions

In the physical prototype experiment, the mobile robot can pass through the narrow gap between obstacles stably and safely, and can run correctly according to the direction indicated by the road signs and finally reach the given destination position. The experimental results verify the feasibility of the design, the accuracy of the road sign detection and obstacle avoidance. The method for information fusion of multiple sensors can not only make up for the error generated by a single sensor, but also sense the information of multi-directional and multi-type obstacles of the robot at this moment and realize the obstacle avoidance function. Therefore, it can be widely used in mobile robot systems.

## Figures and Tables

**Table 1:** Parameter list.

Parameter	Meaning
r	The wheel radius
b	Distance between left and right wheel centroid
$w_{l}$	Left wheel speed
$w_{r}$	RPM of the right wheel
$s_{l}$	Slip rate of left wheel
$s_{r}$	The slip rate of the right wheel
α	Sideslip Angle
$\dot{\varphi }$	The yaw velocity of rotation about the z axis in the XOY plane
$v_{Gx}$	The longitudinal velocity of the center of mass
$v_{Gy}$	The lateral velocity of the center of mass

**Table 2:** Notes to physical prototype.

Label	11	111	12	2	22	31	32	33
Name	First mount	Mounting hole	2Nd Mounting block	Rear wheel	Front wheel	IR Sensor	Ultrasonic sensor	Camera

**Table 3:** Actual Distance and test distance.

Actual Distance (y)/cm	Test Distance (x)/cm	Actual Distance (y)/cm	Test Distance (x)/cm	Actual Distance (y)/cm	Test Distance (x)/cm	Actual Distance (y)/cm	Test Distance (x)/cm
10	9.93	35	34.03	60	58.74	85	83.29
15	14.34	40	38.98	65	63.88	90	88.22
20	19.29	45	44.31	70	68.69	95	93.17
25	24.17	50	48.93	75	73.52	100	98.03
30	29.12	55	53.79	80	78.14		

**Table 4:** Actual distance and fitted distance.

Actual Distance /cm	Fitting Distance /cm	Actual Distance /cm	Fitting Distance /cm	Actual Distance /cm	Fitting Distance /cm	Actual Distance /cm	Fitting Distance /cm
10	10.3448	35	34.8545	60	59.9846	85	84.9519
15	14.8298	40	39.887	65	65.2120	90	89.9657
20	19.8639	45	45.3093	70	70.1037	95	94.9999
25	24.8269	50	50.0078	75	75.0158	100	99.9425
30	29.8610	55	54.9504	80	79.7144		

**Table 5:** Experimental data of short distance road sign recognition.

Detection Distance /cm	Number of Inspections	Number of Successful Tests	Number of Errors Detected	Detection Success Rate (Number of Successes/Total Number of Failures)
20	100	63	37	63%
25	100	80	20	80%

**Table 6:** Experimental data of long distance road sign recognition.

Detection Distance /cm	Number of Inspections	Number of Successful Tests	Number of Errors Detected	Detection Success Rate (Number of Successes/Total Number of Failures)
30	100	100	0	100%
40	100	99	1	99%
50	100	100	0	100%
60	100	99	1	99%
70	100	100	0	100%
80	100	99	1	99%
90	100	100	0	100%
100	100	100	0	100%
Average success rate of detection				99.625%

## Data Availability

The data are available upon request.
